# The Salt-Induced Diffusiophoresis of Nonionic Micelles—Does the Salt-Induced Growth of Micelles Influence Diffusiophoresis?

**DOI:** 10.3390/molecules29153618

**Published:** 2024-07-31

**Authors:** Onofrio Annunziata

**Affiliations:** Department of Chemistry and Biochemistry, Texas Christian University, Fort Worth, TX 76109, USA; o.annunziata@tcu.edu

**Keywords:** Triton X-100, sodium chloride, diffusion, preferential hydration, multiple equilibria, micellar distribution, light scattering

## Abstract

Salt-induced diffusiophoresis is the migration of a colloidal particle in water due to a directional salt concentration gradient. An important example of colloidal particles is represented by micelles, generated by surfactant self-assembly in water. For non-ionic surfactants containing polyethylene glycol (PEG) groups, PEG preferential hydration at the micelle–water interface is expected to drive micelle diffusiophoresis from high to low salt concentration. However, micelles are reversible supramolecular assemblies, with salts being able to promote a significant change in micelle size. This phenomenon complicates the description of diffusiophoresis. Specifically, it is not clear to what extent the salt-induced growth of micelles affects micelle diffusiophoresis. In this paper, a multiple-equilibrium model is developed for assessing the contribution of the micelle growth and preferential hydration mechanisms to the diffusiophoresis of non-ionic micelles. The available experimental data characterizing the effect of NaCl on Triton X-100 aggregation number are combined with data on diffusiophoresis and the preferential hydration of PEG chains to show that the contribution of the micelle growth mechanism to overall diffusiophoresis is small compared to that of preferential hydration.

## 1. Introduction

The transport properties of colloidal particles in water are important for many technologies [1,2,3], including microfluidics [4,5,6,7,8], purification [6,7,9,10], coating [11,12], enhanced oil recovery [3,5,13,14], drug delivery [15,16,17] and detergency [17,18]. One way to induce the migration of particles in aqueous fluids is by applying directional concentration gradients of salts [8,19,20,21,22]. This transport mechanism, known as diffusiophoresis [23,24], has attracted much attention because it promotes particle focusing [8], and separation [6,7], controlled release [15], deposition [25,26], water purification [9,27] and hydrocarbon extraction [28]. Many studies on diffusiophoresis have focused on colloidal particles that are electrically charged [6,8,9,19,20,22,26]. In this case, diffusiophoresis is electrophoretic in nature; it originates from the internal electric field produced by salts that possess cation and anion with appreciably different mobilities (e.g., NaCl) [8,20,24,29,30,31]. However, diffusiophoresis can also occur for neutral particles, such as polyethylene glycol (PEG) and nanoparticles coated with PEG motifs. Consistent with this hypothesis, diffusiophoresis coefficients of PEG chains in the presence of salts have been reported [21,32]. In this case, diffusiophoresis is caused by the preferential hydration of PEG [21,33]. Since salt increases PEG chemical potential, PEG diffusiophoresis occurs from high (low) to low (high) salt (water) concentration because PEG thermodynamically prefers water to salt.

Diffusiophoresis can also occur in the case of supramolecular aggregates such as micelles [34,35,36]. These are colloidal particles produced by the reversible self-assembly of surfactant molecules in water occurring above the critical micelle concentration (cmc) [37]. Due to their ability to host nonpolar molecules, micelles find applications in detergency [38], extraction [39], catalysis [40] and as carriers for the delivery of therapeutic agents [41,42]. Clearly, understanding the diffusiophoresis of micelles is also relevant to the manipulation of small guest molecules in the technological applications listed above. For example, micelle diffusiophoresis could be exploited for the extraction of non-polar molecules from dead-end pores [28,43], relevant to enhancing oil recovery [13] and soil remediation [39]. A well-known category of surfactants is that of hydrocarbon lipophilic groups covalently linked to PEG chains. These nonionic surfactants are commonly found in the household, as well as in the chemical and pharmaceutical industries [44].

**Figure 1:**
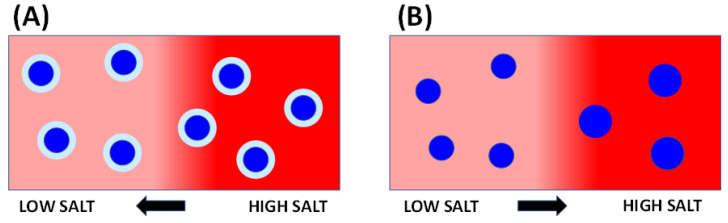
(**A**) The preferential hydration mechanism of micelle diffusiophoresis in the presence of salt concentration gradient. High (**right**) and low (**left**) salt concentrations are shown as a red color contrast for simplicity. Micelles are shown as blue spherical particles for simplicity, surrounded by a light-blue layer enriched with water due to preferential hydration. Arrow indicates diffusiophoresis direction: micelle migration occurs from the high to the low salt concentration due to the preference of micelles for water compared to salt. (**B**) The micelle growth mechanism of micelle diffusiophoresis. Micelles are shown as spherical particles for simplicity, with small and large sizes at low and high salt concentrations, respectively. Arrow indicates diffusiophoresis direction: since small micelles diffuse faster than large micelles, there is a net diffusiophoresis from the low to the high salt concentration.

Diffusiophoresis studies on PEG chains are important for understanding the diffusiophoresis of PEG-based neutral micelles. Indeed, it is expected that the salt-induced diffusiophoresis of their micelles is also driven by PEG preferential hydration [36]. As illustrated in Figure 1A, the layer surrounding the micelle surface is enriched with water molecules compared to the salt–water bulk fluid. Preferential hydration [45] causes the chemical potential of micelle to increase with salt concentration and drives their migration toward a low salt concentration. 

Interestingly, salts also thermodynamically affect PEG-based micelles in other ways. Indeed, they are known to reduce cmc and increase micelle size [46,47]. The salt-induced growth of micelles may be caused by a corresponding reduction in repulsive interactions [48,49,50] between PEG chains on the micelle surface due to a decrease in solvent quality, occurring especially in the case of strong salting-out agents. Consistent with this, it is also known that salts promote liquid–liquid phase separation in aqueous PEG mixtures [51]. Thus, a reversible reorganization of micellar assemblies along a salt gradient occurs. This phenomenon can affect micelle diffusiophoresis [35]. The hypothesized effect of salt-induced micelle growth on diffusiophoresis is illustrated in Figure 1B. Here, smaller and larger micelles are located at low and high salt concentrations, respectively. Since smaller micelles diffuse faster than larger micelles, there will be a net diffusion of micelles from low to high salt concentration. Since surfactant concentration in Figure 1B is uniform along the salt gradient, this is a diffusiophoretic mechanism [35]. Note that this phenomenon is further enhanced when fluid viscosity increases with salt concentration, because diffusion decreases as viscosity increases according to the Stokes–Einstein equation [52]. 

The micelle growth mechanism is predicted to generate diffusiophoresis in the direction opposite to that caused by preferential hydration. Thus, it is important to understand the extent to which it contributes to the overall diffusiophoresis, especially for those surfactants that undergo a significant increase in micelle size in the presence of salt.

Experimental diffusiophoresis studies have been previously reported on tyloxapol [35,36], a PEG-based surfactant that is essentially an oligomer of Triton X-100 [53,54,55]. It was found that the salt-induced diffusiophoresis of tyloxapol micelles is closely related to that of PEG chains, thereby showing that the diffusiophoresis of PEG-based particles is governed by the interaction between PEG moieties on the micelle surface and surrounding fluid. Furthermore, measurements of dynamic light scattering (DLS) have shown that the hydrodynamic radius of tyloxapol micelles remains approximately constant up to ionic strengths as high as 1 M in the presence of Na_2_SO_4_ [36], a strong salting-out agent. At higher ionic strengths, the micelle radius starts to significantly increase. Nonetheless, a model based on chemical equilibrium between two micellar aggregates was used to assess that the contribution of micelle growth to diffusiophoresis remains small compared to preferential hydration. 

In contrast to the tyloxapol case, DLS measurements on Triton X-100 in water have shown that the micelle hydrodynamic radius steadily increases with NaCl concentration [46]. Correspondingly, light-scattering intensity data showed that the Triton X-100 aggregation number of ≈100 in water more than doubled its value at the NaCl ionic strength of 1 M. Remarkably, even the behavior of the Tyloxapol micelle diffusion coefficient in water is different from that of Triton X-100 micelles. In the Tyloxapol case, it increases with surfactant concentration, as expected in the presence of repulsive interactions between micelles [17]. In contrast, the diffusion coefficient of Triton X-100 micelles decreases as surfactant concentration increases [46]. This is consistent with a concentration-induced micelle growth [56]. This suggests that salt-induced micelle growth is more significant for Triton X-100 than tyloxapol micelles.

In this paper, a novel multiple-equilibrium model describing the salt-induced diffusiophoresis of nonionic micelles is developed. Experimental data describing the effect of NaCl on the Triton X-100 average aggregation number [46] are used to quantify the micelle growth mechanism. On the other hand, experimental data characterizing the effect of NaCl on diffusiophoresis and the preferential hydration of free PEG chains [21] are used to quantify the preferential hydration mechanism. It will be shown that the contribution of the micelle growth mechanism remains small compared to that of preferential hydration mechanism.

## 2. Theory

### 2.1. Description of Micelle Diffusiophoresis

Micelle diffusiophoresis can be introduced through the following isothermal and isobaric linear law: [20,57,58,59]

(1) $$v_{P}=-D_{P}\;\left(\nabla \ln C_{P}+{\hat{D}}_{\mathrm{PS}}\;\frac{\nabla \mu_{S}}{RT}\right)$$

where $v_{P}$ is the net diffusion rate of micelle particles (P) in the solvent-fixed reference frame [58,59,60,61]. $D_{P}$ is the micelle Brownian mobility (or tracer-diffusion coefficient), $C_{P}$ is the surfactant molar (or mass) concentration and $\mu_{S}$ is the salt (S) chemical potential, with R and T being the ideal gas constant and absolute temperature, respectively. The values of $D_{P}$ are experimentally determined by DLS [62], Taylor dispersion [63] and interferometry [64]. DLS has been extensively used to measure the diffusion coefficients of micelles [65,66]. Typically, the collective (or mutual) diffusion coefficient of micelles is measured as a function surfactant concentration above cmc. The extrapolation of diffusion coefficients to cmc yields the value of $D_{P}$. Note that the pulsed field gradient NMR can also be used to extract the $D_{P}$ of micelles [67]. However, since this technique yields self-diffusion coefficients, the contribution of free surfactant to the measured diffusion coefficient is known to be significant, unless cmc is very low. In the NMR case, $D_{P}$, can also be evaluated by adding highly hydrophobic probes that are entirely incorporated by the micelles [68,69].

In Equation (1), the reduced diffusiophoresis coefficient, ${\hat{D}}_{PS}$, is introduced. This characterizes the relative magnitude of diffusiophoresis compared to micelle mobility, $D_{P}$ [59,70]. Note that $\nabla \mu_{S}$ represents the thermodynamic driving force responsible for diffusiophoresis and is connected to salt concentration, $C_{S}$, through $\nabla \mu_{S}/RT=\nu_{S}y_{S}/C_{S}$, with $\nu_{S}$ being the number of ions in the salt formula ($\nu_{S}=2$ for NaCl) and $y_{S}$ the known thermodynamic factor characterizing thermodynamic non-ideality of the binary salt–water system, with $y_{S}\to 1$ when $C_{S}\to 0$ [21,71,72]. It is important to note that this description also applies to neutral cosolutes such as osmolytes, for which $\nu_{S}=1$ [58]. The term $\nabla \ln C_{P}$ in Equation (1) represents the restoring Brownian entropic force. This description of diffusiophoresis is analogous to that of electrophoresis [57,73,74], where an external electrical-potential gradient causes the migration of charged particles.

The diffusiophoresis coefficient, ${\hat{D}}_{\mathrm{PS}}$, is then formally split into two contributions:

(2) $${\hat{D}}_{\mathrm{PS}}={\hat{D}}_{\mathrm{PS}}^{\left(h\right)}+{\hat{D}}_{\mathrm{PS}}^{\left(m\right)}$$

where ${\hat{D}}_{\mathrm{PS}}^{\left(h\right)}$ and ${\hat{D}}_{\mathrm{PS}}^{\left(m\right)}$ are the preferential hydration (superscript, h) and micelle growth (superscript, m) contributions to overall diffusiophoresis, respectively. Our goal is to derive mathematical expressions for both ${\hat{D}}_{\mathrm{PS}}^{\left(h\right)}$ and ${\hat{D}}_{\mathrm{PS}}^{\left(m\right)}$.

### 2.2. Effect of Salt on Micellization Thermodynamics

To describe the effect of salt (or any cosolute in general) on the thermodynamics of micellization, it is assumed that total surfactant concentration, $C_{P}$, is low enough that micelle–micelle interactions can be neglected. As in previous works [48,49,50], free surfactant is in chemical equilibrium with an ensemble of micellar aggregates with aggregation number m=2, 3, 4,… The mass fraction of surfactant in the micelle of aggregation number m is $X_{m}$, with $X_{m}\;C_{P}/m$ representing the molar concentration of this specific micellar species. Clearly, the set of $X_{m}$ represents a mass distribution of surfactant, with $X_{1}$ being the fraction of free surfactant. The set of $X_{m}$ is subjected to the normalization condition (mass conservation): $\sum_{m=1}^{\infty }X_{m}=1$. Its differentiation yields:

(3) $$\sum_{m=1}^{\infty }dX_{m}=0$$

Chemical equilibrium between micelles and free surfactant [48,50] is described by

(4) $$X_{m}=me^{-g_{m}}{X_{1}}^{m}{C_{P}}^{m-1}$$

where $g_{m}\equiv (\mu_{m}^{0}-m\mu_{1}^{0})/RT$ is a reduced standard Gibbs free energy of micellization (with $g_{1}\equiv 0$), with $g_{m}/m$ representing the free energy change per surfactant unit. Note that the value of cmc, which conventionally represents the free surfactant concentration at $X_{1}\;=\;1/2$, is equal to ${\left(e^{g_{m}}/m\right)}^{1/(m-1)}$ in the case of monodisperse micelles with aggregation number m [49]. In general, $g_{m}$ is a function of the salt–water composition but is independent of $C_{P}$ (although its value does depend on the chosen concentration scale for $C_{P}$). We shall assume that $g_{m}$ is a function of salt osmolarity, $\pi_{S}$. This is directly related to salt chemical potential through the Gibbs–Duhem relation: $d\pi_{S}=\;[C_{S}/(1-C_{S}{\bar{V}}_{S})]\;d\mu_{S}/RT$ or $d\pi_{S}=\nu_{S}y_{S}dC_{S}/(1-C_{S}{\bar{V}}_{S})$. When $y_{S}\approx 1$, osmolarity is approximately the total concentration of ions in solution. Clearly, salt enhances micellization if $g_{m}$ decreases as $\pi_{S}$ increases.

Two other important thermodynamic factors describing micellizations, $y_{m}$ and $K_{m}$, are introduced below. Specifically, the differentiation of $X_{m}(C_{P},\pi_{S})$ in Equation (3) gives:

(5a) $$y_{m}\equiv 1+{\left(\frac{\partial \ln X_{m}}{\partial \ln C_{P}}\right)}_{\pi_{S}}=m\left[1+{\left(\frac{\partial \ln X_{1}}{\partial \ln C_{P}}\right)}_{\pi_{S}}\right]$$

(5b) $$K_{m}\equiv {\left(\frac{\partial \ln X_{m}}{\partial \pi_{S}}\right)}_{C_{P}}=G_{m}+m{\left(\frac{\partial \ln X_{1}}{\partial \pi_{S}}\right)}_{C_{P}}$$

where $G_{m}\equiv -dg_{m}/d\pi_{S}$, with the negative sign ensuring that $G_{m}$ is positive when salt enhances micellization, and pressure and temperature subscripts typically appended to these partial derivatives are omitted to alleviate notation. As in the case of $g_{m}$, $G_{m}$ also increases with m. The insertion of Equations (5a) and (5b) into Equation (3) shows that

(6a) $$y_{1}=\frac{1}{<m>}$$

(6b) $$K_{1}=-\frac{<G_{m}>}{<m>}$$

where $<a>\;\equiv \sum_{m=1}^{\infty }aX_{m}/\sum_{m=1}^{\infty }X_{m}$. These expressions of $y_{1}$ and $K_{1}$ can be then used to rewrite Equations (5a) and (5b) in the following way:

(7a) $$y_{m}=\frac{m}{<m>}$$

(7b) $$K_{m}=G_{m}-\frac{m}{<m>}<G_{m}>$$

with $<y_{m}>\;=1$ and $<K_{m}>\;=0$. In the presence of salt-induced micelle growth, $K_{m}$ is positive (negative) when m is larger (smaller) than <m>.

To explicitly characterize salt-induced micelle growth, we can take the derivative of $\sum_{m=1}^{\infty }mX_{m}$ with respect to $\pi_{S}$ and use Equation (4) to obtain:

(8) $$m_{S}^{\prime }\equiv {\left(\frac{\partial \ln<m>}{\partial \pi_{S}}\right)}_{C_{P}}=\frac{<mG_{m}>-<m><G_{m}>}{<m>}-p<G_{m}>$$

where $(<mG_{m}>-<m><G_{m}>)/<m>$ must be a positive parameter characterizing how $G_{m}$ increases with m, and p is the polydispersity parameter:

(9) $$p\equiv \frac{<m^{2}>-<m{>}^{2}}{<m{>}^{2}}$$

The micelle growth parameter, $m_{S}^{\prime }$, will be linked to ${\hat{D}}_{\mathrm{PS}}^{\left(m\right)}$ in Equation (2).

### 2.3. Preferential Hydration Diffusiophoresis

Each micellar aggregate is subjected to diffusiophoresis as described by Equation (1). For a micelle with aggregation number m, we have:

(10) $$v_{m}=-D_{m}\left[\nabla \ln(C_{P}X_{m})+{\hat{D}}_{m\;S}^{\left(h\right)}\;\frac{\nabla \mu_{S}}{RT}\right]$$

where $X_{m}C_{P}$ characterizes the surfactant concentration of this specific micelle, $v_{m}$ its net diffusion rate, $D_{m}$ its Brownian mobility and ${\hat{D}}_{m\;S}^{\left(h\right)}$ its intrinsic diffusiophoretic coefficient, which is attributed to preferential hydration. As already shown in Figure 1A, ${\hat{D}}_{m\;S}^{\left(h\right)}$ characterizes micelle diffusiophoresis from high to low salt concentration. Note that Equation (10) also includes the case of free surfactant (m=1) for completeness.

According to the preferential hydration mechanism, we can write:

(11) $${\hat{D}}_{m\;S}^{\left(h\right)}=h_{m}{\left(\frac{\partial \mu_{m}}{\partial \pi_{S}}\right)}_{C_{P}}\frac{C_{S}}{1-C_{S}{\bar{V}}_{S}}$$

where $h_{\;m}<1$ is a positive hydrodynamic coefficient, which has been previously described using a hydrodynamic model [70] and a two-domain model [58,59], and $\mu_{m}$ is the chemical potential of the micellar aggregate. The quantity, ${\left(\partial \mu_{m}/\partial \pi_{S}\right)}_{C_{P}}$, is a preferential interaction coefficient [45,59,75], characterizing the effect of salt on micelle chemical potential. It is positive if salt increases micelle chemical potential, i.e., when preferential hydration occurs. Note that ${\left(\partial \mu_{m}/\partial \pi_{S}\right)}_{C_{P}}$ is a molar volume; it essentially represents a volumetric layer characterizing the excess of water surrounding a micelle. We can then write:

(12) $${\left(\frac{\partial \mu_{m}}{\partial \pi_{S}}\right)}_{C_{P}}=\nu_{m}\;{\bar{V}}_{W}$$

where ${\bar{V}}_{W}$ is the water partial molar volume ($18.07\;{\mathrm{cm}}^{3}\cdot {\mathrm{mol}}^{-1}$ at 25 °C) and $\nu_{m}$ is the number of water molecules characterizing solvent thermodynamic excess near the micelle [59]. The hydrodynamic coefficient, $h_{\;m}$, can be interpreted as the fraction of $\nu_{m}$ water molecules outside the micelle slip boundary, with $1-h_{\;m}$ being the fraction of $\nu_{m}$ that diffuses together with the micelle [58,59].

### 2.4. Micelle Growth Diffusiophoresis

The coefficient, ${\hat{D}}_{m\;S}^{\left(h\right)}$, does not fully characterize diffusiophoresis alone because $X_{m}$ in $\nabla \ln(C_{P}X_{m})$ of Equation (10) is, in general, also a function of salt chemical potential or osmolarity. If we assume that chemical equilibrium between micellar aggregates is fast compared to micelle diffusion and diffusiophoresis [34,35], we can apply thermodynamic relations locally. Thus, $\ln(C_{P}X_{m})$ can be differentiated with respect to $C_{P}$ and $\pi_{S}$, yielding:

(13) $$\nabla \ln(X_{m}C_{P})=y_{m}\nabla \ln C_{P}+K_{m}\;\frac{C_{S}}{1-C_{S}{\bar{V}}_{S}}\frac{\nabla \mu_{S}}{RT}$$

where the second term, which is proportional to $\nabla \mu_{S}/RT$, represents the micelle growth contribution to diffusiophoresis. Accordingly, we set:

(14) $${\hat{D}}_{m\;S}^{\left(m\right)}\;=K_{m}\frac{C_{S}}{1-C_{S}{\bar{V}}_{S}}$$

So that we can rewrite Equation (10) in the following way:

(15) $$v_{m}=-D_{m}\;\left[y_{m}\nabla \ln C_{P}+({\hat{D}}_{m\;S}^{\left(m\right)}+{\hat{D}}_{m\;S}^{\left(h\right)})\frac{\nabla \mu_{S}}{RT}\right]$$

According to Equation (14), the sign of ${\hat{D}}_{m\;S}^{\left(m\right)}$ is directly related to that of $K_{m}$. This means that small micelles ($K_{m}<0$), which are expected to be located in the low-salt concentration side along the salt gradient, tend to diffuse toward high salt concentration. On the other hand, large micelles ($K_{m}>0$) in the high-salt concentration side diffuse in the opposite direction. This is consistent with the illustration in Figure 1B.

### 2.5. Role of Multiple Equilibrium on Micelle Brownian Mobility and Diffusiophoresis

In the two previous subsections, the effect of salt on diffusiophoresis of a specific micelle with aggregation number m was examined by introducing expressions for ${\hat{D}}_{m\;S}^{\left(m\right)}$ and ${\hat{D}}_{m\;S}^{\left(h\right)}$. In this section, the expression of the overall diffusiophoresis coefficients, ${\hat{D}}_{PS}^{\left(h\right)}$ and ${\hat{D}}_{PS}^{\left(m\right)}$ in Equation (2), will be derived, taking into account the presence of multiple micellar aggregates in chemical equilibrium with each other. 

To link Equation (15) to Equation (1), we observe that the total surfactant flux, $C_{P}v_{P}$, must be the sum of all fluxes, $\sum_{m=1}^{\infty }C_{P}X_{m}v_{m}$, based on mass conservation. This implies the net migration rate of micelles, $v_{P}$, is given by the mean:

(16) $$v_{P}=\;<v_{m}>$$

Insertion of Equation (15) into (16) gives the expression of Brownian mobility:

(17) $$D_{P}=\;<y_{m}D_{m}>\;=\frac{<mD_{m}>}{<m>}$$

where Equation (7a) was used for $y_{m}$. It is expected that $D_{m}$ decreases as m increases, i.e., salt-induced micellar growth leads to a decrease in the value of $D_{P}$. It is also important to note that the contribution, $D_{m}$, of a specific micellar aggregate is weighted by $mX_{m}$. This is why the contribution of free surfactant diffusion coefficient ($D_{1}$) to $D_{P}$ can be usually neglected above cmc. For example, if we apply Equation (17) to free surfactant at $X_{1}\approx 1\%$ in equilibrium with micelles with aggregation number of m≈100 and $D_{1}/D_{m}\approx 10$, one calculates that $D_{m}$ is just 0.1% lower than $D_{P}$. Remarkably, this consideration is not only true for light-scattering measurements but for any experimental technique that yields mutual-diffusion coefficients [76]. Indeed, it is a result of the chemical equilibrium, not a light-scattering *z*-average of diffusion coefficients. 

In relation to diffusiophoresis, the insertion of Equation (15) into (16) yields:

(18a) $${\hat{D}}_{PS}^{\left(m\right)}=\;\frac{<D_{m}\;{\hat{D}}_{m\;S}^{\left(m\right)}>}{D_{P}}=\frac{<D_{m}\;K_{m}>}{D_{P}}\frac{C_{S}}{1-C_{S}{\bar{V}}_{S}}\approx \frac{<D_{m}\;K_{m}>}{D_{P}}C_{S}$$

(18b) $${\hat{D}}_{PS}^{\left(h\right)}=\;\frac{<D_{m}\;{\hat{D}}_{m\;S}^{\left(h\right)}>}{D_{P}}=\frac{<D_{m}\;h_{m}\;\nu_{m}>{\bar{V}}_{W}}{D_{P}}\frac{C_{S}}{1-C_{S}{\bar{V}}_{S}}\approx \frac{<D_{m}\;h_{m}\;\nu_{m}>{\bar{V}}_{W}}{D_{P}}C_{S}$$

where we have also used Equation (14) for ${\hat{D}}_{PS}^{\left(m\right)}$ and Equations (11) and (12) for ${\hat{D}}_{PS}^{\left(h\right)}$. Note that both diffusiophoresis coefficients are directly proportional to salt concentration, $C_{S}\approx C_{S}/(1-C_{S}{\bar{V}}_{S})$, because usually $C_{S}{\bar{V}}_{S}<<1$. For example, $C_{S}{\bar{V}}_{S}=0.020$ for NaCl at 1 M [20,77].

In the expression of ${\hat{D}}_{PS}^{\left(m\right)}$, it is important to assess the sign of $<D_{m}\;K_{m}>$, since $K_{m}<0$ for small micelles (m lower than <m>) and $K_{m}>0$ for large micelles (m higher than <m>), with $<K_{m}>\;=0$. If $D_{m}$ were a constant independent of m, then $<D_{m}\;K_{m}>\;=0$, thereby implying that micelle growth diffusiophoresis is absent in this limiting case. However, it is expected that $D_{m}$ appreciably decreases as m increases. This implies that the negative contribution of $D_{m}\;K_{m}$ for small micelles is more important than the positive contribution of $D_{m}\;K_{m}$ for large micelles. Thus, we can conclude that $<D_{m}\;K_{m}>$ and ${\hat{D}}_{PS}^{\left(m\right)}$ are negative. On the other hand, all $D_{m}\;h_{m}\;\nu_{m}$ terms in Equation (18b) are positive so that ${\hat{D}}_{PS}^{\left(h\right)}$ is also positive, with the preferential hydration mechanism invariably driving micelle diffusiophoresis from a high to low salt concentration.

## 3. Discussion

**Figure 2:**
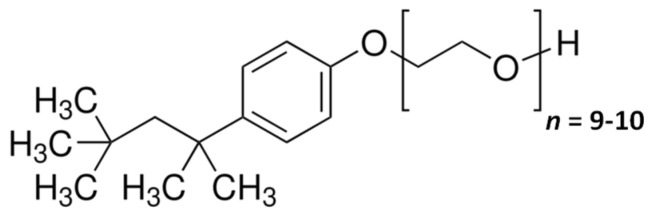
Chemical structure of Triton X-100, with the hydrophilic PEG group (9–10 ethoxy units) covalently attached to the hydrophobic 4-(1,1,3,3-tetramethylbutyl)-phenyl group.

In this section, experimental results on aqueous Triton X-100 [46,47] and free PEG chains [21] in the presence of NaCl at 25 °C are used to evaluate preferential hydration and micelle growth contributions to micelle diffusiophoresis. The chemical structure of Triton X-100 is shown in Figure 2. 

Experimental studies on micelles are often carried out in a concentration range well above cmc, where $X_{1}<<1$. Light-scattering experiments on aqueous Triton X-100 were carried out at surfactant concentrations not lower than ≈10 g·L^−1^, while cmc is 0.15 g·L^−1^ in water and decreases in the presence of NaCl [46]. This implies that $X_{1}$ is of the order of $X_{1}\approx 0.01$ or less. Thus, ignoring free surfactant leads to errors of the order of ≈1% in the evaluation of the mean value, <m>, by static light scattering. As will be shown below, ${\hat{D}}_{PS}^{\left(m\right)}$ and ${\hat{D}}_{PS}^{\left(h\right)}$ are both proportional to micelle aggregation number, <m>, consistent with diffusiophoresis being an interfacial phenomenon. This means that, as in the case of <m>, the contribution of free surfactant can also be neglected when examining ${\hat{D}}_{PS}^{\left(m\right)}$ and ${\hat{D}}_{PS}^{\left(h\right)}$. From now on, we shall assume that diffusiophoresis is evaluated in conditions in which $X_{1}<<1$.

**Figure 3:**
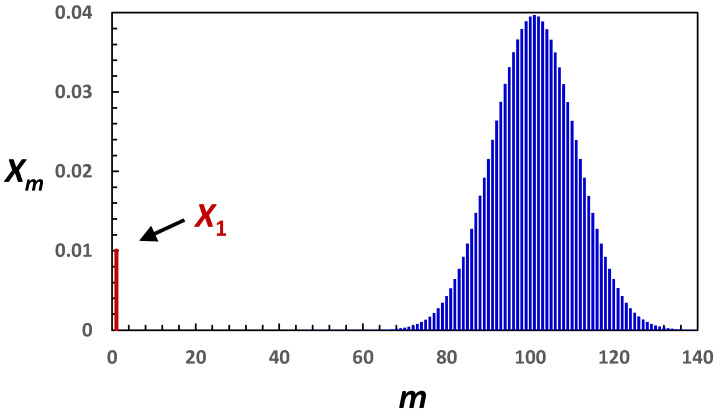
Representative mass distribution of spherical micelles. Mass fraction of surfactant, $X_{m}$, as a function of micelle aggregation number m, calculated using $X_{1}=0.01$ and assuming that $X_{m}=(1-X_{1})(m/m_{0})e^{-{\left(m-m_{0}\right)}^{2}/2\sigma^{2}}/\sqrt{2\pi \sigma^{2}}$, with $m_{0}=100$ and $\sigma =\sqrt{m_{0}}=10$ (p=0.01). Note that $<m>\;=m_{0}(1+\sigma /m_{0})\approx m_{0}$ and $<m^{2}>-<m{>}^{2}\;=\sigma^{2}(1-\sigma^{2}/{m_{0}}^{2})\approx \sigma^{2}$ from Gaussian integrals. This quasi-Gaussian distribution function can be derived from Equation (4), assuming that Gibbs-free energy of micellization is approximated by its second-order series expansion, $g_{m}=g_{m0}+g_{m0}^{\prime }(m-m_{0})+g_{m0}^{\prime \prime }{\left(m-m_{0}\right)}^{2}/2$, with $g_{m0}$, $g_{m0}^{\prime }$ and $g_{m0}^{\prime \prime }$ being $g_{m}$, $dg_{m}/dm$ and $d^{2}g_{m}/dm^{2}$ at $m=m_{0}$, and $m_{0}$ being the aggregation number at the maximum of micelle concentration, $X_{m}/m$. At $m=m_{0}$, $d(X_{m}/m)/dm=\ln(X_{1}C_{P})-g_{m0}^{\prime }=0$, consistent with previous work [50].

For many surfactants, including Triton X-100, we can assume that <m> >>1. For example, the Triton X-100 aggregation number is 105 in water and becomes 230 in 1 M NaCl [46]. This surfactant produces approximately spherical micelles in water [78,79]. This type of micelle is predicted to possess a fairly narrow distribution width, with polydispersity parameter, p (see Equation (9)), approximately equal to 1/<m> [56]. For example, if <m> = 100, then the distribution width is ≈10 and p≈0.01. Thus, the contribution of micellar aggregates with less than m≈50 and more than m≈150 is negligible. This case is portrayed by the representative mass distribution in Figure 3.

As discussed in Section 2, knowledge of how $D_{m}$ varies with m is important for characterizing micelle growth diffusiophoresis. For a narrow range of micellar aggregates, we can assume that micelle diffusion coefficient $D_{m}$ linearly decreases as m increases:

(19) $$D_{m}=D^{0}-D^{\prime }m$$

where $D^{0}$ and $D^{\prime }$ are positive constants. Note that $D^{0}$ is a diffusion coefficient that carries no particular physical interpretation, while $D^{\prime }$ is directly relevant to micelle growth diffusiophoresis because it describes the extent to which small micelles diffuse faster than large micelles.

It is important to note that narrow distribution widths are predicted for spherical micelles [56]. However, salt-induced micellar growth is likely to produce more elongated cylindrical micelles, with a distribution width that is relatively large compared to that of spherical micelles [49,80]. The experimental dependence of $D_{P}$ on <m> will be used to examine the validity of Equation (19). 

To describe the dependence of $D_{P}$ on <m>, Equation (19) is inserted into Equation (17) to obtain:

(20) $$D_{P}=D^{0}-(1+p)D^{\prime }<m>$$

To apply Equation (20), experimental data of <m> and $D_{P}$, extracted from light-scattering experiments at 25 °C on Triton X-100, were taken from Ref. [46]. The values of $\pi_{S}$, which will be needed to extract $m_{S}^{\prime }$, were calculated from corresponding NaCl concentrations using thermodynamic data in Ref. [71].

**Figure 4:**
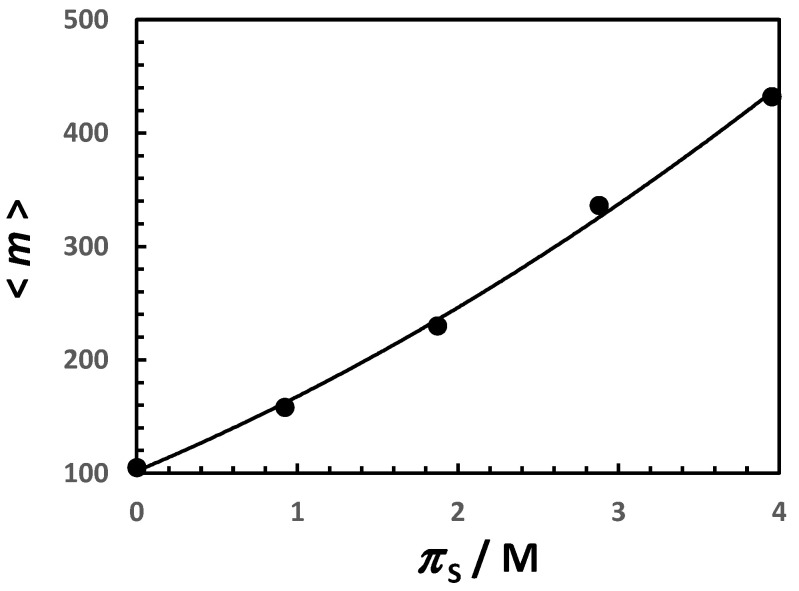
Average aggregation number, <m>, as a function of NaCl osmolarity, $\pi_{S}$, at 25 °C. Values of <m> were taken from Ref. [46]. Values of $\pi_{S}$ were calculated from thermodynamic data in Ref. [71]. Solid linear curve is a quadratic fit through the data.

**Figure 5:**
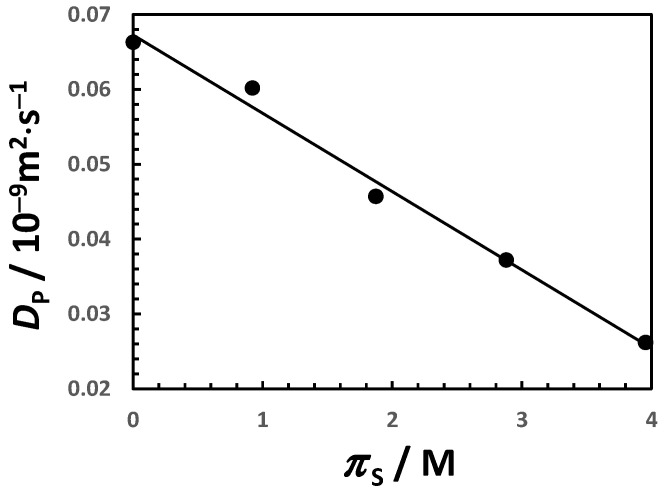
Micelle diffusion coefficient, $D_{P}$, as a function of NaCl osmolarity, $\pi_{S}$, at 25 °C. Values of $D_{P}$ were taken from Ref. [46]. Values of $\pi_{S}$ were calculated from thermodynamic data in Ref. [71]. Solid linear curve is a linear fit through the data.

The average micelle aggregation number for aqueous Triton X-100 as a function of NaCl osmolarity is shown in Figure 4. Starting from <m> ≈ 100 in water, it undergoes more than a 4-fold increase when NaCl osmolarity reaches 4 M (i.e., $C_{S}$ ≈ 2 M). In Figure 5, the micelle diffusion coefficient linearly decreases as salt osmolarity increases, reducing to 40% of its value in water at $\pi_{S}$ = 4 M. This is mostly related to a 2-fold increase in the micelle hydrodynamic radius, with a relatively small contribution (≈15%) coming from the increase in solution viscosity [81].

**Figure 6:**
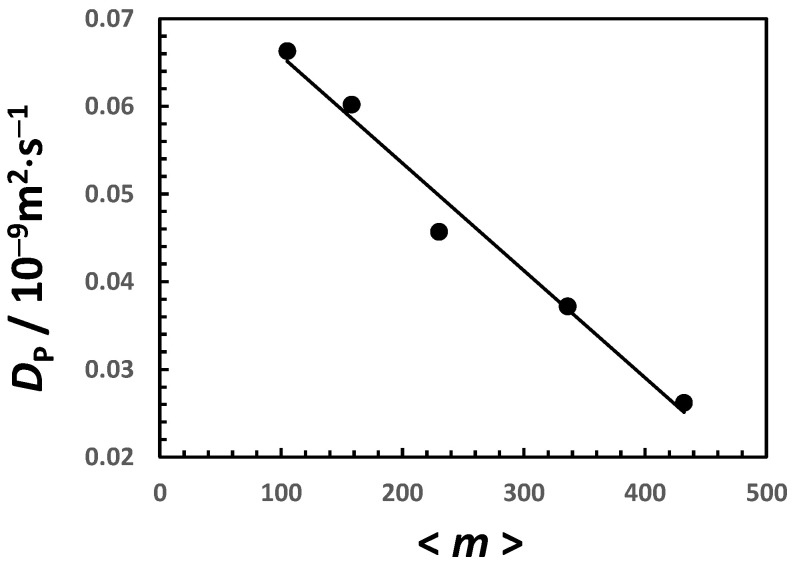
Micelle diffusion coefficient, $D_{P}$, as a function of average aggregation number, <m>. Values of $D_{P}$ and <m> were taken from Ref. [46]. Solid linear curve is a linear fit through the data.

Data in Figure 4 and Figure 5 are combined to generate the plot of $D_{P}$ as a function of <m>  in Figure 6. The observed linear decrease in $D_{P}$ as <m>  increases is consistent with $(1+p)D^{\prime }$ in Equation (18) being a constant within the experimental error. This indicates that either p is a constant or p<<1. The method of least squares is then applied to extract $(1+p)D^{\prime }=1.22\times 10^{-13}\;m^{2}\cdot s^{-1}$.

To examine how micelle polydispersity affects the interpretation of the slope in Figure 5, it is important to appreciate that $(1+p)D^{\prime }$ is greater than $D^{\prime }$. Thus, the extracted slope represents the upper limit to the value of $D^{\prime }$. It is also important to observe that p<<1 and $(1+p)D^{\prime }\approx D^{\prime }$ if p≈1/<m> is used to describe the case of spherical micelles. However, p might not be negligible for cylindrical micelles. To further examine the significance of micelle polydispersity, Equation (8) may be used together with the experimental data of <m> in Figure 4. Consistent with models applied to cylindrical micelles, we hypothesize that the behavior of $G_{m}$ is described by the linear relation, $G_{m}=G_{m_{.0}}+G_{m_{.0}}^{\prime }\cdot (m-m_{0})$ with $m\geq m_{0}$, where $m_{0}$ is the taken to be the average aggregation number of micelles in water, $G_{m_{.0}}\equiv G_{m}(m_{.0})$, and $G_{m_{.0}}^{\prime }\equiv dG_{m}/dm$ at $m=m_{0}$ is a positive constant characterizing how salt favors micelle growth. The substitution of this expression of $G_{m}$ into Equation (7b) leads to $K_{m}=(G_{m_{.0}}^{\prime }m_{0}-G_{m_{.0}})(m/<m>-1)$ and Equation (8) leads to $m_{S}^{\prime }=(G_{m_{.0}}^{\prime }m_{0}-G_{m_{.0}})p$, where $G_{m_{.0}}^{\prime }m_{0}-G_{m_{.0}}$ is a positive constant. This noteworthy result implies that $m_{S}^{\prime }$ is directly proportional to p. Experimental values of $m_{S}^{\prime }$, which are extracted from the curve in Figure 4, are reported in Table 1 as a function of NaCl concentration (and osmolarity). Here, we can see that $m_{S}^{\prime }$ decreases as $\pi_{S}$ increases, thereby indicating that p also decreases. Thus, if it is assumed that p<<1 for micelles in water, then this approximation should remain valid in the presence of salt. This analysis, together with the linear behavior of $D_{P}$ in Figure 6, supports the hypothesis that Equation (19) is valid and p<<1 in Equation (20).

We are now in position to use Equation (19) for $D_{m}$ and Equation (7b) for $K_{m}$ to rewrite $<D_{m}\;K_{m}>$ in Equation (18a) in the following way:

(21) $$<D_{m}\;K_{m}>\;=D^{\prime }<m\;K_{m}>\;=-\;D^{\prime }\;\left(<m\;G_{m}>-\frac{<m^{2}>}{<m>}<G_{m}>\right)$$

This leads to the expression of micelle growth diffusiophoresis:

(22) $${\hat{D}}_{PS}^{\left(m\right)}\approx \;-<m>m_{S}^{\prime }\frac{D^{\prime }}{D_{P}}C_{S}$$

This equation shows that ${\hat{D}}_{PS}^{\left(m\right)}$ is negative, as illustrated in Figure 1B. It is proportional to the micelle aggregation number, <m> , as expected for an interfacial phenomenon, $m_{S}^{\prime }$, which characterizes salt-induced micelle growth, and $D^{\prime }/D_{P}$, which describes how micelle mobility decreases with aggregation number [23].

We now turn our attention to preferential hydration diffusiophoresis as described by Equation (18b). It is expected that the larger the number of hydrophilic groups (e.g., PEG) on micelle surface, the larger thermodynamic excess of water molecules, $\nu_{m}$, is. Thus, the value of $\nu_{m}$ is directly proportional to the micelle aggregation number, m. In relation to the hydrodynamic coefficient, $h_{m}$, the experimental results on the diffusiophoresis of free PEG chains and tyloxapol PEG-based micelles allow us to deduce that $h_{m}$ is a weak function of particle shape [21,36,59]. As previously mentioned, this transport parameter can be interpreted as the fraction of $\nu_{m}$ water molecules that are outside the slip boundary of the diffusion micelle. Consistent with this observation, $h_{m}$ in Equation (11) can be assumed to be a constant, independent of m. This makes the diffusiophoresis coefficient, ${\hat{D}}_{m\;S}^{\left(h\right)}$, directly proportional to m, which is again consistent with diffusiophoresis being an interfacial phenomenon. The higher the micelle surface area, the larger preferential hydration diffusiophoresis is. In summary, we can write:

(23a) $$\nu_{m}=m\;\bar{\nu }$$

(23b) $$h_{m}=\;\bar{h}$$

where $\bar{\nu }$ is the water thermodynamic excess per surfactant unit in a micelle, while the hydrodynamic coefficient is assumed to be a constant $\bar{h}$, independent of m. Substituting this in Equation (18b) yields:

(24) $${\hat{D}}_{PS}^{\left(h\right)}\;\approx \;<m>\;\bar{\nu }\bar{h}\;{\bar{V}}_{W}C_{S}$$

Previous diffusiophoresis experiments on Tyloxapol micelles have shown that $\bar{\nu }$ and $\bar{h}$ are approximately the same as those extracted from the diffusiophoresis experiments on PEG chains [21,36] Specifically, the value of $\bar{\nu }$ per ethoxy group is 2.4 water molecules for one ethoxy group of PEG in the presence of NaCl. Since each Triton X-100 surfactant contains ≈10 ethoxy groups (see Figure 2), the value of $\bar{\nu }\approx 24$ can be used per surfactant unit in a micelle. The value of $\bar{h}\approx 0.14$ does not significantly change from PEG to Tyloxapol micelles and can also be used for Triton X-100 micelles [21]. This can be interpreted as 14% of $\bar{\nu }\approx 24$ water molecules outside the slip boundary of the micelle.

We are now in position to compare magnitude of preferential hydration diffusiophoresis with micelle growth diffusiophoresis. The values of ${\hat{D}}_{PS}^{\left(m\right)}$ and ${\hat{D}}_{PS}^{\left(h\right)}$ were calculated using Equations (22) and (24), respectively, and reported in Table 2. The data in Table 1, together with $D^{\prime }\approx D^{\prime }(1+p)=1.22\times 10^{-13}\;m^{2}\cdot s^{-1}$, can be used to calculate ${\hat{D}}_{PS}^{\left(m\right)}$. Note that ${\hat{D}}_{PS}^{\left(m\right)}$ values would be lower if p were not negligible. 

In Table 2, we can appreciate that the magnitude of ${\hat{D}}_{PS}^{\left(m\right)}$ is about 1–2% that of ${\hat{D}}_{PS}^{\left(h\right)}$. This noteworthy result shows that preferential hydration is the dominant diffusiophoresis mechanism, even if salt produces a significant change in micelle aggregation number as in the case of Triton X-100. This is mostly related to the moderate reduction in $D_{P}$ as <m>  increases. In other words, it is possible to neglect the effect of salt on micellization and assume that salt-induced micelle diffusiophoresis is caused by preferential hydration alone.

## 4. Conclusions

The diffusiophoresis coefficient of a nonionic micelle, ${\hat{D}}_{PS}$, is the sum of two contributions, ${\hat{D}}_{PS}^{\left(m\right)}$ and ${\hat{D}}_{PS}^{\left(h\right)}$, which are caused by salt-induced micelle growth and preferential hydration, respectively. According to the model developed in this paper, both contributions are proportional to the average micelle aggregation number, <m> , but have opposite sign, with ${\hat{D}}_{PS}^{\left(m\right)}<0$ and ${\hat{D}}_{PS}^{\left(h\right)}>0$. The coefficient, ${\hat{D}}_{PS}^{\left(m\right)}$, is proportional to $m_{S}^{\prime }$, describing salt-induced micelle growth, and is also proportional to the transport parameter, $D^{\prime }/D_{P}$, which characterizes the extent to which micelle growth reduces Brownian mobility. On the other hand, the coefficient ${\hat{D}}_{PS}^{\left(h\right)}$ is a function the thermodynamic excess of water molecules, $\bar{\nu }$, which characterizes the preferential hydration of the surfactant PEG chain in the presence of salt. It is also proportional to the transport parameter, $\bar{h}$, which can be interpreted as the fraction of $\bar{\nu }$ molecules outside the slip boundary of the diffusing micelle. All parameters were evaluated for Triton X-100 in the presence of aqueous NaCl from available experimental data at 25 °C. Data analysis shows that the contribution of ${\hat{D}}_{PS}^{\left(m\right)}$ is small compared to that of ${\hat{D}}_{PS}^{\left(h\right)}$ for the Triton X-100 micelle. In other words, PEG-based micelles, when undergoing salt-induced diffusiophoresis, can be approximately treated as fixed colloidal particles, i.e., the reorganization of these supramolecular assemblies along the salt gradient does not significantly influence micelle diffusiophoresis. This work provides the theoretical basis for quantifying the contribution of micelle growth and preferential hydration mechanisms to the diffusiophoresis of nonionic micelles in the presence of salt gradients. This is crucial for the interpretation of experimental results on micelle diffusiophoresis. Future theoretical studies should examine the contribution of the micelle growth mechanism to the salt-induced diffusiophoresis of ionic micelles.

## Figures and Tables

**Table 1:** Effect of NaCl on thermodynamic micellization parameters of Triton X-100.

$C_{S}/M$	$\pi_{S}/M$	<m>	$m_{S}^{\prime }/M$
0	0	102	0.58
0.20	0.369	125	0.51
0.50	0.921	162	0.44
1.00	1.873	235	0.35

**Table 2:** Transport parameters for Triton X-100.

$C_{S}/M$	$D_{P}/10^{-9}\;m^{2}\cdot s^{-1}$	$D^{\prime }/D_{P}$	${\hat{D}}_{PS}^{\left(m\right)}/C_{S}/M^{-1}$	${\hat{D}}_{PS}^{\left(h\right)}/C_{S}/M^{-1}$
0	0.0678	0.0018	−0.11	6.19
0.20	0.0636	0.0019	−0.12	7.57
0.50	0.0574	0.0021	−0.15	9.84
1.00	0.0471	0.0026	−0.21	14.29

## Data Availability

No new experimental data were created in this study. The data analysis presented in this study is available on request from the corresponding author.
